# Evaluation of the *Good Start Program*: a healthy eating and physical activity intervention for Maori and Pacific Islander children living in Queensland, Australia

**DOI:** 10.1186/s12889-016-3977-x

**Published:** 2017-01-13

**Authors:** Seema Mihrshahi, Lisa Vaughan, Nicola Fa’avale, Shreenika De Silva Weliange, Inez Manu-Sione, Lisa Schubert

**Affiliations:** 1 Faculty of Medicine and Biomedical Sciences, School of Public Health, The University of Queensland, Brisbane, 4006 Australia; 2 Prevention Research Collaboration, Sydney Medical School & Sydney School of Public Health, The University of Sydney, The Charles Perkins Centre D17, Level 6, The Hub, Sydney, NSW 2006 Australia; 3 SHORE and Whariki Research Centre, College of Health, Massey University, Auckland, New Zealand; 4 Faculty of Medicine, Department of Community Medicine, University of Colombo, Colombo, Sri Lanka; 5 Good Start Program, Child and Youth Community Health Service, Children’s Health Queensland Hospital and Health Service, Spring Hill, Brisbane, 4006 Australia; 6 School of Professional Studies and Education, Griffith University, Mount Gravatt, Brisbane, 4122 Australia

**Keywords:** Evaluation, Maori and Pacific Islander, Interventions to reduce obesity, Obesity, Children, Healthy eating, Physical activity

## Abstract

**Background:**

Reducing the prevalence of obesity and chronic disease are important priorities. Maori and Pacific Islander communities living in Australia have higher rates of obesity and chronic disease than the wider Australian population. This study aims to assess the effectiveness of the Good Start program, which aims to improve knowledge, attitudes and practices related to healthy eating and physical activity amongst Maori and Pacific Islander communities living in Queensland.

**Methods:**

The intervention was delivered to children aged 6–19 years (*N =* 375) in schools by multicultural health workers. Class activities focused on one message each term related to healthy eating and physical activity using methods such as cooking sessions and cultural dance. The evaluation approach was a quantitative uncontrolled pre-post design. Data were collected each term pre- and post-intervention using a short questionnaire.

**Results:**

There were significant increases in knowledge of correct servings of fruit and vegetables, knowledge of sugar and caffeine content of common sugar-sweetened drinks, recognition of the consequences of marketing and upsizing, and the importance of controlling portion size (all *P <* 0.05). There was also increases in knowledge of physical activity recommendations (*P <* 0.001), as well as the importance of physical activity for preventing heart disease (*P <* 0.001) and improving self-esteem (*P <* 0.001). In terms of attitudes, there were significant improvements in some attitudes to vegetables (*P =* 0.02), and sugar-sweetened drinks (*P <* 0.05). In terms of practices and behaviours, although the reported intake of vegetables increased significantly (*P <* 0.001), the proportion of children eating discretionary foods regularly did not change significantly, suggesting that modifying the program with an increased emphasis on reducing intake of junk food may be beneficial.

**Conclusion:**

The study has shown that the Good Start Program was effective in engaging children from Maori and Pacific Island backgrounds and in improving knowledge, and some attitudes and practices, related to healthy eating and physical activity. The evaluation contributes valuable information about components and impacts of this type of intervention, and considerations relevant to this population in order to successfully change behaviours and reduce the burden of chronic disease.

## Background

Obesity, one of the most important risk factors of nutrition-related chronic disease, is a growing global pandemic. In Australia, around one in four children aged 5–17 are overweight or obese, comprised of 20.2% overweight and 7.4% obese [1]. Reducing this prevalence is at the core of most preventive health strategies for nutrition-related chronic disease in Australia. Amongst ethnic minority groups there are some, including Maori and Pacific Islander communities, that have a particularly high prevalence of chronic disease including obesity and Type 2 Diabetes and are at a higher risk of hospitalisation for these diseases [2]. There is also evidence that obesity is significantly more prevalent among children from Pacific Islander backgrounds [3]. In New Zealand for example, 15% of Maori and 30% of Pacific children aged 2–14 years were obese compared to the national average of 11% [4].

Maori and Pacific Islander people migrated to Australia from the island groups of Micronesia, Melanesia, Polynesia, with the majority migrating from New Zealand. They are referred to collectively as ‘Maori and Pacific Islander people’, and despite often being grouped together, the cultures are heterogeneous with diverse customs, languages and religions. While diversity exists, some of the cultural values and beliefs among Maori and Pacific Islander groups are similar. This includes a strong foundation on the family relationship and a strong religious affiliation. Food and feasting is of particular cultural importance and traditionally, in the Pacific Islands cultures, larger stature has been associated with beauty, social standing, health and wealth [5]. Maori and Pacific Islanders also have a high degree of cultural connection through music, songs and dance and have documented and disseminated their history and traditions using these methods [6, 7], so over time, Maori and Pacific Island cultures have become known for their love of music, dance and performing arts [7, 8]. Designing interventions to address chronic disease in Maori and Pacific Islander population will need to take into account these cultural values.

Reducing the prevalence of obesity in Maori and Pacific Islander communities has become an urgent priority, particularly in the state of Queensland in Australia where the population of Maori and Pacific Islanders is growing and estimated to comprise at least two percent of the Queensland population [9]. Numerous dietary intake studies conducted in Maori and Pacific Island Countries have illustrated the connection between changing nutritional choices at the population and individual level and rising obesity levels [5]. A needs assessment conducted by the Queensland State Government [2] identified poor dietary habits, poor nutritional understanding and a lack of culturally tailored health promotion as the major issues contributing to obesity and chronic disease for the Maori and Pacific Islander community. The Good Start Program was developed in response to this needs assessment.

Good Start is a community based program for Pacific Islander and Maori children, delivered primarily through schools, with the engagement of families and community organisations. The intervention is composed of a number of strategies built around key messages focused on improving nutrition and increasing physical activity. Class activities focused on one message every school term using innovative and culturally appropriate methods. It was identified very early on in the program that presenting nutrition and physical activity content using ‘Western’ teaching methods was not appropriate for engaging Maori and Pacific Islander children or families, and that using their traditional tools of song and dance would be a more effective method of delivery. Hence, all sessions contained an element of performing arts. The school environment is a pragmatic setting for interventions that target children for a number of reasons, most obviously, that they spend a considerable proportion of their time there [10]. It is also an important time for their development as they are starting to form peer groups that may influence their eating habits and attitudes to, and participation in, sport and physical activity. In this paper we describe the Good Start intervention and the results of the first year of evaluation and some lessons learned as part of the intervention and evaluation planning.

## Methods

The Good Start Program is a community-based program aiming to improve knowledge, attitudes and practices related to healthy eating and physical activity amongst Maori and Pacific Islander communities living in Queensland. More information about the program as a whole is available online [11]. This manuscript describes the activities undertaken in the school based program and evaluates these activities.

### Schools intervention design

The main objectives for the school based activities were promotion of vegetables, decreasing consumption of sugar-sweetened drinks, decreasing unhealthy snacks and reducing portion size and increasing physical activity. Messages reinforcing each objective were presented using performing arts in a fun and interactive way including chanting, music, dance and theatre. Ice-breakers and games were used to review previously covered information and role plays were used to present new concepts. Modified rap songs and chants reinforced key messages. Table 1 gives an overview of the methods used for nutrition education and physical activity in the schools as part of the Good Start Program.

**Table 1:** Main activities included in the Good Start Program to convey key messages

Activity	Description	Development/Preliminary feedback
Challenge Book	A simple diary distributed to students to monitor their daily physical activity and nutrition intake. A student shades in a check box relating to specific lifestyle behaviour choices (e.g. drink a sugary drink, eat vegetables, exercise, watch TV).	This helps the students to become more aware of their lifestyle choices and see progressive changes they have made. Some teachers have found this to be a particularly useful tool and have incorporated it into their curriculum planning.
MPI Can You Dance?	A creative blending of hip hop and cultural dance with healthy lifestyle information. It incorporates street dance, contemporary style, cultural dance and current music to engage children and explore ideas surrounding healthy living.	By weaving in cultural dance, it creates a sense of individual, community and cultural pride, while educating children on the importance of healthy lifestyle choices. It was developed in partnership with a local community organisation working with MPI youth.
MPI Eat Well	A classroom based nutrition education program based on the four key messages. Children learn nutrition and physical activity content through interactive games and fun activities.	This program is delivered during class time and the information presented is at a slightly more advanced level and students are supported to build a deeper understanding of health. Some schools have acknowledged the comprehensive nature of the information and include it as part of their health curriculum.
MPI Got Talent	Students create a role play scenario that promotes healthy lifestyle behaviours.	The MHWs work with the students to prepare a short performance ensuring the content of messages is accurate. Role plays tend to use a variety of performance styles from cultural dance, singing, chanting and acting.
MPI Junior Chef	The aim of this activity is to enable each child to develop basic cooking skills and build basic nutrition knowledge. The MPI Junior Chef utilises culturally tailored resources such as the ‘Healthy Taro Leaf’ and modified family recipes. MPI Junior Chef also incorporates the use of a ‘Kitchen Licence’ system. This system progressively builds the children’s skills as their cooking experience increases.	Kids enjoy a sense of achievement as they progress from a Basic Licence to an MPI Junior Chef.
Power Up	Designed for high school students this structured one hour session is broken into two components: a nutrition segment and a physical activity segment. Each session is highly interactive with the use of many practical demonstrations, props, visual and audio media, personal stories and testimonies.	Within MPI communities it is these types of communication strategies that are most effective in conveying messages. This unique culture and environment has received positive comments from many of the school staff and principals.
Power Up Leadership	This program was implemented with high school students for the purpose of training and developing future MPI Leaders to lead the way for change in their school, family and community. Students with potential leadership qualities (or those who might benefit from some additional structure and support) are identified by the school or selected by the MHWs. Weekly lunchtime meetings are delivered covering cultural leadership principles, group interactions and discussions with MPI guest speakers.	This program aims to build the capacity of students to take on responsibilities and lead change.
Island Flavour	The Island Flavour program is based on traditional MPI games. The games showcased are physically active and fun. Each week students “travel” to different islands using a passport in which they write down the key learning from each island, e.g. “60 min everyday, got to keep on moving”	The Island Flavour sessions were very well received by students and teachers. They provide an excellent opportunity to learn about health, physical activity and MPI cultural heritage.

### Intervention delivery

The intervention was delivered by trained multicultural health workers (MHWs) who were also responsible for cultural tailoring and building community relationships. In alignment with Queensland Health Multicultural Guidelines [12], all of the MHW’s were of Maori or Pacific Islander descent and lived within the communities they serviced, including representatives from seven communities: Cook Islands, Fiji, Fiji Indian, Maori, Samoan, Tongan and Papua New Guinean. The target groups for the program were children of Maori and Pacific Island background attending school. The program activities were implemented in schools with high numbers of children from Maori and Pacific Islander backgrounds (ranging from 10-90% of the total school population) and a total of nineteen public schools in Queensland received the intervention. All schools were in the lower quarters of the Index of Community Socio-Educational Advantage [13].

### Evaluation planning and data collection

In order to assess changes in knowledge attitudes and practices relating to healthy eating and physical activity, a pre-post test design was used. A participatory approach was used to develop a protocol for the evaluation involving the program manager, nutritionists, co-ordinator, MHW’s and other staff. This helped to ensure that the evaluation was also of value to the staff and the logistics and feasibility of the data collection were acceptable to the staff who had a significant role in explaining the evaluation purpose and tools, and gathering the data.

### Questionnaires

For the pre and post intervention questionnaires, all children from schools who took part in the program were invited to participate and parents were informed via distribution of Participant Information Sheets addressed to parents/guardians and given to children to take home. The program manager was available to answer any questions that parents had before deciding whether or not to provide written consent. Children who were absent on the day of data collection and children who did not have written consent from their parent to participate in the evaluation were excluded from the evaluation. The initial method of the pre and post questionnaires was self-administered, but after feedback from the MHWs about the length of time needed and the complexity of the questionnaires particularly for younger children, an interviewer administered approach was taken. The questionnaires were interviewer administered by trained public health nutrition students and the MHWs. Training protocols and interviewer guides were developed to ensure that data was collected in a consistent manner across multiple sites. The questions were incorporated from the *‘Eat Well be Active’* community programs [14] and the Australian National Nutrition and Physical Activity Survey [1] which have been validated in Australian children. Some additional questions assessing key learnings specific to the program were also incorporated. Questions about age, sex, culture and school were also included on each questionnaire.

*Knowledge and practices regarding fruit and vegetables* Knowledge of fruit and vegetables recommendations was assessed by asking: How many serves of fruit/vegetables should you eat everyday? (response categories for each from 0 to 6). Children were asked to identify whether certain vegetables were starchy or non-starchy. Each response to knowledge questions were coded as either 1 (correct) or 0 (incorrect answer).

Children were also asked about their practices: How many serves of fruit/vegetables do you usually eat each day? (with a picture of a serve to indicate quantity and response categories from 0 to 6). Adequate intakes were coded as 1 with inadequate intakes as 0.

*Knowledge and practices regarding snack foods, portion size and sugar-sweetened beverages* Knowledge was assessed using true or false responses to a series of statements listed in Table 4 and 5. These included statements relating to the bodies preferred fuel source, eating mindfully, salt sugar and fat content of processed snack foods, amounts of sugar and caffeine in commonly consumed drinks and consequences of sugar and caffeine consumption. Responses were coded as either 1 (correct) or 0 (incorrect answer).

Validated food frequency questions were used to assess intakes of snacks and discretionary foods (response categories never, less than once a week, 1–3 times week, 4 to 6 times a week or every day). These were dichotomized to 3 times or less a week or and 4 times a week or more.

*Knowledge and practices regarding physical activity* Children were asked about their knowledge of physical activity recommendations and relationship between physical activity and heart disease, dental disease and self-esteem. Responses were coded as either 1 (correct) or 0 (incorrect answer).

Children were asked the number of times per week they did certain activities and the usual amount of times they spend doing these activities on each occasion. Activities were grouped into organised sports (such as rugby league, soccer, netball, touch football, basketball, volleyball, or other) and non organised (activities such as walking, riding a bike, running, kicking a ball or any other non-organised activity).

*Attitudes to healthy eating and physical activity* Children were given a list of statements to agree or disagree with on a 5 point likert scale (from strongly agree to strongly disagree). Responses were dichotomized and strongly agree and agree were coded as 1, and not sure, disagree and strongly disagree were coded as 0. Items were reverse coded where necessary. The list of statements is given in Tables 3 to 6.

### Data analysis

Data from the pre and post questionnaire was entered into IBM SPSS Statistics version 22 for analysis. Descriptive statistics were reported as means (±SD) for continuous measures and proportions (%) for categorical measures. Chi-squared tests were used to determine differences in knowledge, attitudes and practices pre- and post-intervention. Odds ratios (with 95% confidence intervals) giving the estimate of change in knowledge, attitudes and practices as a result of the intervention were reported after adjustment for age and gender. Differences were regarded significant at the *P <* 0.05 level.

## Results

The evaluation of the Good Start Program covered a period of one year from July 2013 to June 2014. A total of 19 schools and approximately 375 children were engaged in the school-based activities and participated in the evaluation, however there were some term-by-term variations in participation numbers.

Table 2 shows the age, gender and cultural background of children participating in the Good Start program. The mean age of the children was 11.0 (SD 2.5) years and ages ranged from 6 to 19 years. The largest proportions of children were from Samoan (24%), Maori (21%) or mixed Islander (37%) cultural backgrounds.

**Table 2:** Age, gender and cultural background of participants in the Good Start Program (Term 1, *N =* 375)

Characteristic		*N*	%
Age	5–11 years	254	67.8
	12–19 years	114	30.4
	Missing	7	1.9
Gender	Male	183	48.8
	Female	187	49.9
	Missing	5	1.3
Cultural Background	Samoan	89	23.7
	Maori	79	21.1
	Cook Islander	27	7.2
	Tongan	16	4.3
	PNG	13	3.5
	Fijian	5	1.3
	Mixed background/other	139	37.1
	Missing	7	1.9

Table 3 shows the results of the evaluation relating to the intervention involving fruits and vegetables. There was a significant increase in knowledge of the correct serves of fruit and vegetables, and in attitudes to vegetables. Most notably there was an increase in reported intake of vegetables with 15% reporting that they ate the recommended five serves of vegetables at the beginning of the term, increasing to 27% by the end of the term. The proportion of children eating the recommended intake of two serves of fruit was already high pre-intervention (88%) and increased non-significantly to 92% after the intervention.

**Table 3:** Changes in knowledge, attitudes and practices regarding fruits and vegetables

Fruit and Vegetable	Question/Statement	% with correct answer Pre-test (95% CI) *N =* 375	% with correct answer Post-test (95% CI) *N =* 369	*P* value	AOR^a^ for change	*P* value
Knowledge	How many serves of vegetables should you eat every day	50.5 (46.1–56.0)	61.7 (56.5–66.8)	0.002	1.58 (1.17–2.14)	0.003
	How many serves of fruit should you eat every day	59.2 (54.2–64.7)	68.1 (63.1–72.9)	0.010	1.53 (1.12–2.08)	0.007
	Sweet potato is which type of vegetable (starchy/non-starchy)	75.6 (71.2–80.2)	80.8 (76.4–84.8)	0.090	1.43 (0.98–2.09)	0.062
	Lettuce is which type of vegetable (starchy/non-starchy)	68.6 (63.9–72.5)	76.0 (71.3–80.4)	0.030	1.49 (1.04–2.09)	0.028
		% with strongly agree/agree Pre-test	% with strongly agree or agree Post-test			
Attitudes	Eating vegetables makes me feel healthy	89.1 (85.5–92.1)	93.6 (90.6–95.7)	0.030	1.64 (0.95–2.82)	0.073
	I like the taste of many vegetables	65.0 (59.9–69.9)	73.3 (68.4–77.8)	0.020	1.45 (1.05–2.07)	0.024
	It is easy to prepare vegetables to eat	76.2 (71.5–80.4)	82.5 (92.0–96.8)	0.040	1.56 (1.07–2.27)	0.020
	Eating fruit makes me feel healthy	94.8 (92.0–96.8)	96.1 (93.5–97.8)	0.410	1.55 (0.719–3.33)	0.264
	I like the taste of most fruits	92.8 (89.7–95.3)	94.7 (91.8–96.8)	0.300	1.58 (0.82–3.00)	0.165
	Fruit is an easy snack	94.8 (92.0–96.9)	94.7 (91.8–96.8)	0.940	1.14 (0.56–2.30)	0.719
		% with adequate intake Pre-test	% with adequate intake Post-test			
Practices	How many serves of vegetables do you usually eat each day	14.9 (11.4–18.9)	26.8 (22.2–31.7)	<0.001	2.06 (1.41–3.02)	<0.001
	How many serves of fruit do you usually eat each day	88.4 (84.7–91.5)	92.2 (88.9–94.8)	0.080	1.51 (0.91–2.51)	0.114

^a^AOR = Adjusted Odds Ratio for change from pre- to post-test, adjusted for age and gender of the children

Table 4 shows knowledge, attitudes and practices regarding sugar-sweetened drinks. Pre-intervention knowledge of amounts of sugar in commonly consumed drinks was low (41%). There were significant increases in knowledge in a number of concepts for this intervention including knowledge of sugar content and consequences of high sugar intake. There were also significant improvements in attitudes to soft drinks, sports drinks and energy drinks as a result of the intervention.

**Table 4:** Changes in knowledge and attitudes regarding sugar-sweetened drinks

Soft drinks	Question/Statement	% with correct answer Pre-test *N =* 421	% with correct answer Post-test *N =* 326	*P* value	AOR^a^ for change	*P* value
Knowledge	How many teaspoons of sugar are there in a 600 mL coke	40.9% (36.0–45.4)	84.0%(79.3–87.5)	<0.001	8.34 (5.73–12.15)	<0.001
	Too much sugar in the diet can contribute to obesity	64.0% (59.9–69.0)	90.5%(86.9–93.4)	<0.001	5.36 (3.47–8.28)	<0.001
	Refined carbohydrates are a good source of vitamins and minerals (neg)	15.5% (13.1–20.2)	53.5%(48.7–59.5)	<0.001	5.79 (4.08–8.20)	<0.001
	Energy drinks have high caffeine and sugar content	69.8% (66.1–74.8)	87.3%(83.4–90.8)	<0.001	2.78 (1.86–4.16)	<0.001
	Being overweight or obese increases your risk of developing diabetes	63.7%(60.0–69.2)	86.5%(82.8–90.3)	<0.001	3.58 (2.42–5.30)	<0.001
	Soft drink marketing encourages people to drink more soft drinks	55.7%(52.2–61.6)	79.8%(75.5–84.3)	<0.001	3.00 (2.13–4.21)	<0.001
	Caffeine in energy drinks is good for children’s developing brain and heart (neg)	70.6% (64.6–73.4)	86.5%(79.7–87.8)	<0.001	3.35 (2.18–5.12)	<0.001
	Upsizing is one of the techniques used to get people to buy larger sizes	43.1%(40.0–49.4)	76.7%(72.6–81.8)	<0.001	4.17 (3.00–5.78)	<0.001
		% with strongly disagree/disagree Pre-test	% with strongly disagree/disagree Post-test			
Attitudes	I enjoy drinking soft drinks (neg)	29.1%(25.7–34.4)	42.2%(37.3–48.1)	<0.001	1.49 (1.14–2.00)	0.009
	I drink soft drinks because my friends drink soft drinks (neg)	15.0% (11.9–18.7)	9.8%(7.0–13.5)	0.060	0.53 (0.33–0.86)	0.010
	I think water is boring (neg)	17.1 (13.5–20.7)	14.1 (10.3 = 17.9)	0.270	0.79 (0.52–1.19)	0.261
	I like to drink energy drinks (neg)	51.9%(47.1–56.7)	63.0% (57.6–68.2)	0.002	1.60 (1.81–2.17)	0.002
	Energy drinks give me energy to study in school or play sport (neg)	51.6%(46.8–56.4)	64.5%(59.1–69.6)	<0.001	1.65 (1.22–2.24)	0.001
	I like to drink sports drinks because all the sports stars drink them (neg)	62.7%(58.0–67.3)	76.0%(71.1–80.5)	<0.001	1.95 (1.40–2.74)	<0.001

(neg) = those who disagreed or strongly disagreed with this statement

^a^AOR = Adjusted Odds Ratio for change from pre- to post-test, adjusted for age and gender of the children

Table 5 shows changes in knowledge, attitudes and practices regarding snacks and sugar. A high proportion of children were aware of the link between obesity and chronic disease pre-intervention (83%). After the intervention there was significantly increased recognition that the body’s preferred fuel source was carbohydrate, and that snack foods contribute too much energy to the diet. There was also increased knowledge that reducing portion sizes can help to manage weight and the meaning of ‘eating mindfully’. However attitudes to upsizing did not improve post-intervention. There was significantly increased recognition that buying snacks and lunch out (rather than homemade) can be expensive. With regard to practices there were no significant changes apart from reduced proportions of children consuming potato chips and sweet biscuits and muffins regularly.

**Table 5:** Changes in knowledge, attitudes and practices regarding snacks and sugar

Snacks	Question/Statement	% with correct answer -pre-test *N =* 357	% with correct answer post-test *N =* 305	*P* value	AOR^a^ for change	*P* value
Knowledge	The body’s preferred fuel is carbohydrate	22.8% (18.5–27.6)	53.7% (47.9–59.5)	<0.001	4.18 (2.95–5.92)	<0.001
	Eating mindfully means I am paying attention to what and how much I am eating	57.4% (52.1–62.6)	83.1% (78.4–87.2)	<0.001	3.57 (2.43–5.23)	<0.001
	Processed snack foods are low in salt, sugar and fat (neg)	45.8% (40.5–51.1)	53.6% (47.8–59.4)	0.050	1.00 (0.71–1.43)	0.980
	Being obese means there is a higher chance of getting a chronic disease like diabetes	82.6%(78.5–86.6)	83.8% (79.1–87.7)	0.830	1.05 (0.68–1.62)	0.820
	Chips, lollies, chocolate or take away food are very fatty and give me too much energy	60.8% (55.5–65.9)	68.8% (63.2–74.0)	0.030	1.40 (1.00–1.96)	0.045
	Swapping a large portion size to a smaller one can help to manage your weight	62.2% (56.9–67.2)	79.1% (74.1–83.6)	0.001	2.11(1.48–3.01)	<0.001
	Packing my own lunch box is a great way to control what you eat during the day	75.9% (71.1–80.3)	79.5% (74.5–83.9)	0.300	1.17 (0.80 = 1.72)	0.410
		% with strongly agree or agree – pre-test	% with strongly agree or agree – post-test			
Attitudes	I eat more when I am tired or stressed out	21.1% (16.9–25.7)	27.3% (22.4–32.7)	0.060	1.37 (0.95–1.98)	0.096
	I ask my parents to buy foods or drinks that I see advertised on television	25.8% (21.3–30.6)	24.0% (19.3–29.2)	0.600	0.86 (0.60–1.24)	0.424
	I usually get the largest portion of food on offer, when having take-away food (e.g. upsizing)	21.1%(16.9–25.7)	29.9%(24.8–35.4)	0.009	1.70 (1.18–2.46)	0.004
	I think fruit and vegetable sticks are an easy snack	79.0% (74.5–82.9)	80.9% (76.0–85.1)	0.560	1.10 (0.74–1.63)	0.630
	Buying snacks or lunch out can be very expensive when compared with taking a homemade meal.	60.0% (54.7–65.1)	71.9% (66.4–76.9)	0.002	1.79 (1.27–2.52)	0.001
	I like to snack between meals	47.5% (42.2–52.8)	51.3% (45.5–57.1)	0.250	1.12 (0.82–1.53)	0.490
	I like to eat similar snacks to my friends	31.9% (27.1–37.0)	27.6% (22.7–33.0)	0.230	0.82 (0.58–1.15)	0.254
		% consuming at least 4 times/week pre-test	% consuming at least 4 times/week post-test			
Practices	Packet of chips/snacks (e.g. potato or corn chips twisties)	23.6% (19.3–28.4)	16.1%(12.2–20.7)	0.010	0.58 (0.38–0.87)	0.009
	Chocolate/chocolate bars	9.0% (6.2–12.4)	9.5% (6.5–13.4)	0.890	1.13 (0.65–1.94)	0.663
	Lollies	12.6% (9.4–16.5)	10.9% (7.6–14.9)	0.480	0.87 (0.53–1.41)	0.570
	Muesli bar/ fruit bar/LCM’s	28.7% (24.0–33.7)	29.2% (24.2–34.6)	0.930	0.97 (0.69–1.38)	0.890
	Yoghurt/ custard/chocolate custard	20.0% (16.0–24.5)	20.0% (15.7–24.9)	0.070	0.98 (0.66–1.45)	0.900
	Savoury biscuits/crackers	14.6% (11.1–18.8)	13.8% (10.1–18.2)	0.820	0.94 (0.60–1.47)	0.780
	Sweet biscuits/cake/muffin/doughnut	17.4% (13.6–21.7)	10.9% (7.6–15.0)	0.020	0.59 (0.37–0.94)	0.026
	Ice-cream / ice blocks	24.4% (20.0–29.2)	20.7% (16.3–25.6)	0.260	0.83 (0.57–1.21)	0.342
	Vegetables or salad	65.3% (60.1–70.2)	72.0% (66.6–77.0)	0.060	1.35 (0.96–1.90)	0.085
	Fruit (fresh or canned)	78.6% (74.0–82.7)	74.8% (69.5–79.5)	0.540	0.79 (0.54 –1.15)	0.218
	Dried fruit (e.g. sultanas)	13.4% (10.1–17.4)	15.6%(11.7–20.2)	0.440	1.28 (0.82–1.99)	0.277
	Hot chips/French fries/wedges	15.5% (11.9–19.7)	17.5%(13.4–22.2)	0.530	1.09 (0.71–1.68)	0.700
	Pies/pasties/sausage rolls	18.2% (14.3–22.6)	15.4% (11.5–20.0)	0.350	0.82 (0.53–1.27)	0.380
	Hot dogs	9.2% (6.4–12.7)	9.5%(6.5–13.4)	1.000	0.97 (0.56–1.70)	0.930
	Pizza	12.0% (8.9–15.9)	11.3% (8.0–15.5)	0.810	0.95 (0.58–1.58)	0.850
	Sandwich/roll	63.3% (58.1–68.3)	60.1% (54.3–65.6)	0.420	0.88 (0.63–1.22)	0.440
	Bread/toast	62.4% (56.9–67.2)	58.2% (52.5–63.8)	0.300	0.82 (0.59–1.13)	0.220
	Spaghetti/pasta/noodles/rice	40.3% (35.2–45.6)	39.6% (34.1–45.4)	0.850	0.99 (0.72–1.36)	0.940
	Soup	18.8% (14.9–23.2)	22.3% (17.7–27.5)	0.290	1.29 (0.87–1.91)	0.210

^a^AOR = Adjusted Odds Ratio for change from pre- to post-test, adjusted for age and gender of the children

Table 6 shows the changes in knowledge, attitudes and practices regarding physical activity. Knowledge of the recommendations of physical activity (one hour a day) increased significantly by 45%, from 13% before the intervention to 58% after the intervention. The knowledge that physical activity plays a preventative role in heart disease and improves self-esteem also increased significantly. There was evidence of a shifting of attitudes about fitness and playing sport. Although there were no significant improvements in running, walking, and non-organised sport in total or in organised sports, the proportions of children engaging in all the above activities were higher post-intervention.

**Table 6:** Changes in knowledge, attitudes and practices regarding physical activity

Physical activity	Question/Statement	% with correct answer Pre-test *N =* 361	% with correct answer Post-test *N =* 323	*P* value	AOR^a^ for change	*P* value
Knowledge	PA recommendations (1 h/day)	13.3 (9.7–17.1)	57.6 (52.0–62.7)	<0.001	9.23 (6.31–13.68)	<0.001
	PA prevents heart disease	48.8 (43.5–54.0)	71.8 (66.6–76.7)	<0.001	2.31 (1.66–3.22)	<0.001
	PA prevents dental disease	52.9(47.6–58.2)	61.6 (56.1–66.9)	0.025	1.35 (0.98–1.85)	0.062
	PA improves self esteem	55.7 (50.4–60.9)	72.4 (67.2–77.2)	<0.001	1.94 (1.39–2.70)	<0.001
		% with strongly agree or agree pre-test	% with strongly agree or agree post-test			
Attitudes	I’m just not into sport	10.1 (6.8–13.2)	8.0 (5.1–11.2)	0.416	0.85 (0.49–1.48)	0.572
	Playing sport costs too much	23.5 (18.2–27.1)	24.4 (19.3–28.9)	0.855	1.13 (0.78–1.63)	0.529
	I’m too unfit to play sport	13.9 (10.0–17.2)	8.0 (5.2–11.6)	0.018	0.54 (0.32–0.90)	0.019
	There is no time to do sport	42.7 (36.4–46.8)	35.7(30.4–41.3)	0.067	0.73 (0.53–1.00)	0.060
		% performing the activity regularly pre-test	% performing the activity regularly post-test			
Practices	Running	14.1(10.7–18.2)	17.6 (13.6–22.3)	0.210	1.33 (0.87–2.05)	0.190
	Walking	48.2 (42.9–53.5)	57.3 (51.7–62.7)	0.021	1.34 (0.97–1.83)	0.070
	Organised sport	72.9 (68.0–77.4)	74.3 (70.0–79.0)	0.728	1.01 (0.71–1.43)	0.950
	Non-organised sport	73.7 (68.8–78.2)	79.3 (75.3–83.6)	0.105	1.25 (0.86–1.82)	0.230
	Some activity	91.4 (88.0–94.1)	93.2 (90.5–95.5)	0.395	1.00 (0.55–1.83)	0.980

*PA* Physical activity

^a^AOR = Adjusted Odds Ratio for change from pre- to post-test, adjusted for age and gender of the children

Although there was some loss to follow up between pre and post-tests for most of the intervention terms (ranging from 2-22% depending on the intervention term), there were no significant differences in the age, gender and cultural background between those that completed the study and those who were lost to follow up (all *P >* 0.05).

## Discussion

The study has shown that the Good Start Program was effective in engaging children from Maori and Pacific Island backgrounds. There were significant increases in knowledge as a result of the program, including knowledge of correct serves of fruit and vegetables, knowledge of sugar and caffeine content of common sugar-sweetened drinks, the importance of controlling portion size, knowledge of what mindful eating was and recognition of the consequences of marketing and upsizing. There was also increases in knowledge of physical activity recommendations as well as the importance of physical activity for preventing chronic disease. In terms of attitudes, there were significant improvements in attitudes to vegetables as well as many attitudes related to sugar- sweetened drinks. In terms of practices and behaviours, although the intake of vegetables increased significantly, there were no significant changes in frequency of intakes of most discretionary foods or in overall physical activity.

In recommending modifications to the program, studies show that Pacific Islander populations consume foods normally considered healthy such as fruits, root vegetables and fish which are transformed into less healthy, high-fat foods through preparation methods so an extension to focus on healthy preparation methods may be warranted for older children [5]. In addition, considering the above results, modifying the program with more of a focus on reducing junk food and sugar-sweetened drinks and increasing physical activity may be beneficial. One of the main learnings of the evaluation procedures was that data collection methods and evaluation delivery must be flexible and procedures need to be changed to accommodate the range of reading and comprehension skills in these age groups.

Unique aspects of the program were the way that the intervention messages were conveyed including aspects of dance, rap songs and use of performing arts in general. This is the only evaluation study published to date which is designed to determine the effectiveness of the program messages in Maori and Pacific Islander communities living in Australia. Similar projects to Good Start have been implemented in New Zealand, such as Project Energize, a school based intervention to improve children’s physical activity and nutrition and improve their overall health. This programme and others in New Zealand are much larger than Good Start and not specifically targeted at Maori and Pacific Islanders, although about one third of participants are Maori [15].

In a systematic review of school based obesity prevention programs [16] fourteen studies were evaluated and the most successful interventions were multidisciplinary and involved a mix of physical activity, and educational components. The authors recommended an intervention duration of at least one year and targeting older children who are establishing personal health behaviour patterns. Another meta-analysis examined specific features associated with successful interventions and showed that programs individualised to certain student characteristics such as ethnicity and socio-economic status have better outcomes [17]. These recommended program design elements are present in the Good Start Program.

In an appraisal of the evidence from existing meta-analyses and systematic reviews examining school based programs to prevent and control obesity, recommendations for future studies included publication of more process based outcome measures that include accounts of barriers and challenges encountered when designing and implementing interventions, acceptance measures and evaluations in real life situations [10]. In light of these recommendations we designed a consultative approach to the design of the intervention and evaluation which draws on some of the unique aspects of the Pacific Island culture. Additionally, the program is guided by cultural specific research guidelines to ensure cultural competencies and appropriateness [2, 18].

Strengths of the study are the participatory approach to evaluation involving staff implementing the program (MHWs, nutritionist and program managers) in the evaluation design and in development of the protocols and questionnaires and in giving a cultural interpretation of the results. This input had the advantage of increasing cultural appropriateness of the evaluation, and building capacity and recognition of the importance of evaluation within the staff and influencing perceived ownership of the study. For example, advice from the MHWs was that fruit intake was not generally a problem in Pacific Island communities and hence we are able to refocus the program on the importance of increasing vegetables in the diet. This also explained the increase in self-reported vegetable intake but no increase in self-reported fruit intake as fruit intake was already high pre-intervention.

This study also has some limitations. The activities of the Good Start program were implemented according to the needs and resources of each school and community and thus an evaluation framework with a randomised trial or quasi experimental study was not feasible. Measures such as weights, heights and waist circumference used in some other evaluation studies [19] were not taken due to ethical concerns when working with minority populations. Other limitations include that some of the data was collected by the MHWs who were responsible for implementation of the program, hence this was not a blinded evaluation and may have introduced bias. There was some loss to follow up between pre and post-tests which may have also biased the results, although there was no difference in demographic variables in those that started the study and those lost to follow up. The generalizability of the findings and the evaluation method are limited to Maori and Pacific Islander groups.

## Conclusions

We believe that this study contributes valuable information about the types of interventions which will need to be implemented in these cultural groups in order to improve knowledge of healthy eating and physical activity. There have been inconsistent findings from systematic reviews examining the effectiveness of school programs for reducing childhood overweight and it is clear that innovative and targeted approaches will need to be taken in order to reduce the prevalence of overweight, obesity and their resulting complications in these populations.

## Abbreviations

MPI: Maori and Pacific Islander
PA: Physical activity
PNG: Papua New Guinea
MHW: Multicultural Health Workers
